# Mycobacterium wolinskyi Bloodstream Infection Associated With a Central Venous Port: A Case Report

**DOI:** 10.7759/cureus.96872

**Published:** 2025-11-14

**Authors:** Tomoyuki Araya, Toshiyuki Kita, Takayuki Higashi, Ryo Hara, Hazuki Takato

**Affiliations:** 1 Respiratory Medicine, National Hospital Organization (NHO) Kanazawa Medical Center, Kanazawa, JPN

**Keywords:** device-associated infection, gram-positive rods, maldi-tof mass spectrometry, mycobacterium wolinskyi, rapidly growing mycobacteria

## Abstract

*Mycobacterium wolinskyi* is a rapidly growing non-tuberculous *Mycobacterium* typically associated with surgical wounds or implanted devices. We report an elderly woman with a central venous (CV) port inserted two years earlier for long-term nutritional support after gastric cancer surgery who developed a bloodstream infection caused by *M. wolinskyi*. Gram staining of the positive blood culture revealed slender filamentous Gram-positive rods, prompting suspicion of rapidly growing mycobacteria (RGM). The organism isolated from both blood and port cultures was identified by matrix-assisted laser desorption/ionization-time of flight mass spectrometry. Despite discontinuation of antibiotics due to *Clostridioides difficile* infection, the patient achieved complete recovery following CV port removal alone, with no recurrence during follow-up. This case underscores the need for awareness that *M. wolinskyi* may present as Gram-positive rods on Gram stain and that close collaboration between clinicians and microbiology laboratories is crucial for early recognition and appropriate management of device-associated RGM infections.

## Introduction

*Mycobacterium wolinskyi* is a non-pigmented, rapidly growing non-tuberculous *Mycobacterium* belonging to the *Mycobacterium smegmatis* group, first described in 1999 [1]. In a comprehensive review by Ariza-Heredia et al. [2], 17 cases of *M. wolinskyi* infection were summarized, involving postoperative wounds, bones, prosthetic devices, and the bloodstream in both immunocompetent and immunocompromised patients. Device-associated infections-including prosthetic joint, vascular graft, and automatic implantable cardioverter-defibrillator pocket infections-were particularly common, and most cases required surgical debridement and prolonged combination antimicrobial therapy with favorable outcomes.

Subsequent reports have broadened the clinical spectrum of *M. wolinskyi* infection to include prosthetic joint infections, facial soft-tissue infections following cosmetic procedures, clusters of surgical site infections, prosthetic valve endocarditis, and peritoneal dialysis catheter infections [3-10]. These infections can sometimes lead to severe sepsis or even fatal systemic disease. These observations suggest that *M. wolinskyi* predominantly causes postoperative or device-related infections, and that modern diagnostic tools such as matrix-assisted laser desorption ionization-time of flight mass spectrometry (MALDI-TOF MS) enable rapid and reliable species-level identification.

Here, we report a case of *M. wolinskyi* bloodstream infection associated with an infected central venous (CV) port in an elderly woman. The organism was recovered from both blood and port cultures and subsequently identified by MALDI-TOF MS. We also reviewed the relevant literature to highlight its evolving clinical spectrum and diagnostic challenges.

## Case presentation

An 86-year-old woman with a CV port for long-term intravenous access presented with fever. The CV port had been placed two years earlier for long-term nutritional support due to poor oral intake following surgery for gastric cancer. Fever developed the day before transfer. At the referring hospital, chest-to-pelvic computed tomography (CT) had already been performed, but no definite infectious focus was identified, and a CV port-related infection was suspected. Piperacillin/tazobactam had been started the day before transfer. On admission, the CV port was removed. No abscess formation or purulent discharge was observed around the port site at the time of removal. Antimicrobial therapy was switched to cefazolin thereafter. Laboratory tests showed mild anemia and thrombocytopenia, elevated C-reactive protein suggesting systemic inflammation, mild liver enzyme elevation, and moderate renal impairment, while other results were normal (Table 1).

**Table 1:** Laboratory findings on admission. Laboratory tests on admission showed mild anemia and thrombocytopenia, with markedly elevated CRP levels indicating systemic inflammation. Liver enzymes were mildly elevated, while renal function was moderately impaired. Other parameters were within normal limits. WBC: white blood cell; RBC: red blood cell; Hb: hemoglobin; Ht: hematocrit; Plt: platelet; CRP: C-reactive protein; T-Bil: total bilirubin; TP: total protein; Alb: albumin; ALP: alkaline phosphatase; AST: aspartate aminotransferase; ALT: alanine aminotransferase; LDH: lactate dehydrogenase; Na: sodium; K: potassium; Cl: chloride; BUN: blood urea nitrogen; Cr: creatinine; eGFR: estimated glomerular filtration rate; UA: uric acid.

Parameter	Patient’s value	Reference range
WBC (/µL)	4,900	4,500–9,000
Neutrophil (%)	92.8	38–74
Lymphocyte (%)	3.5	16.5–49.5
Monocyte (%)	3.7	5–10
Eosinophil (%)	0	0–10
Basophil (%)	0	0–2
RBC (×10⁴/µL)	275	382–500
Hb (g/dL)	8.8	11.7–14.6
Ht (%)	25.3	34.3–44.2
Plt (×10⁴/µL)	7.0	15.8–34.8
CRP (mg/dL)	3.29	0–0.4
T-Bil (mg/dL)	0.3	0.3–1.2
TP (g/dL)	6.0	6.7–8.3
ALP (U/L)	55	38–113
AST (U/L)	47	13–33
ALT (U/L)	60	6–27
LDH (U/L)	222	119–229
Alb (g/dL)	3.4	4.0–5.0
Na (mEq/L)	141	135–149
K (mEq/L)	4.2	3.5–4.9
Cl (mEq/L)	107	96–108
BUN (mg/dL)	36.4	8–22
Cr (mg/dL)	1.03	0.5–0.8
eGFR (mL/min/L)	38.7	60–100
UA (mg/dL)	3.1	2.3–7.0
D-dimer (µg/mL)	5.6	0–1

**Figure 1:**
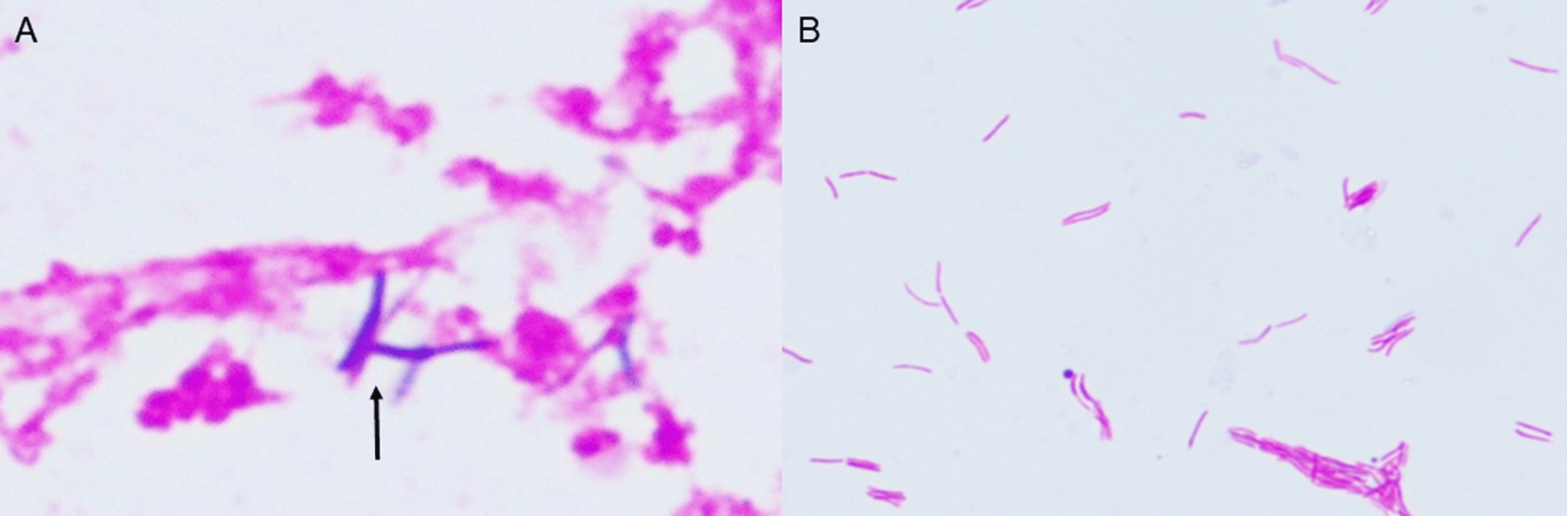
Microscopic findings of Mycobacterium wolinskyi isolated from positive blood culture bottles. (A) Gram staining shows slender, filamentous, Gram-positive rods without branching (arrow) (×1,000, oil immersion). This finding highlights that rapidly growing mycobacteria, including *M. wolinskyi*, can appear as Gram-positive rods in routine Gram staining. (B) Ziehl–Neelsen staining demonstrates slender, acid-fast bacilli without branching (×400).

On hospital day 3, both aerobic blood culture bottles became positive following three days of incubation. Gram staining revealed slender, filamentous, Gram-positive rods, while Ziehl-Neelsen staining of the positive blood culture demonstrated acid-fast bacilli (Figure 1).

**Figure 2:**
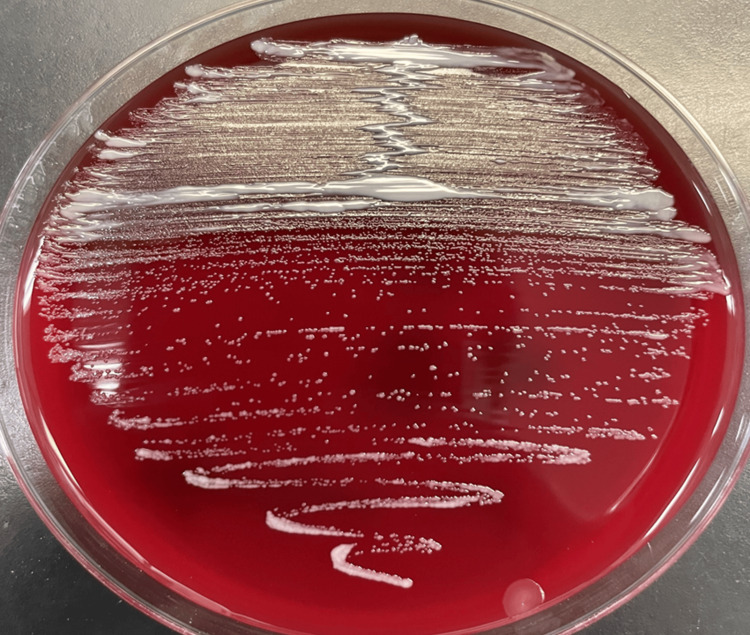
Colonies of Mycobacterium wolinskyi on blood agar. Colonies of *M. wolinskyi* grown on a blood agar plate after 48 hours of incubation at 35°C in our laboratory. The colonies appeared white, flat, round, rough (R-type), and non-hemolytic, suggesting rapidly growing non-tuberculous mycobacteria.

These findings were promptly reported to the attending physician. Subsequent polymerase chain reaction assays performed on the positive blood culture for *Mycobacterium tuberculosis* complex and *Mycobacterium avium* complex were negative, and subcultures from the positive blood culture were placed on blood and chocolate agar plates for further identification. After 24 hours of incubation, only weak growth was observed, but white, rough colonies became visible after 48 hours (Figure 2).

Because of this characteristic morphology, rapidly growing mycobacteria (RGM) were suspected. Ziehl-Neelsen staining performed on colonies obtained from both blood and CV port cultures again demonstrated acid-fast bacilli. Because other potential sources of sepsis could not be completely excluded-as isolated bloodstream infection caused by RGM is uncommon-cefazolin therapy was continued but discontinued on hospital day 7 due to the development of *Clostridioides difficile* infection (CDI). On hospital day 10, MALDI-TOF MS identified the organism as *M. wolinskyi*.

To identify potential sources, cultures from both heel ulcers were also performed, yielding methicillin-resistant *Staphylococcus aureus* but not *M. wolinskyi*. She became afebrile three days after CV port removal and the change of antibiotics. Thereafter, follow-up blood cultures obtained at two-week intervals were negative on two occasions. She continued to improve clinically without recurrence and was discharged after 33 days of hospitalization.

## Discussion

This case highlights *M. wolinskyi* as an uncommon but important cause of bloodstream and device-related infections. Clinicians should be aware that this organism, although acid-fast, may appear as slender, weakly staining Gram-positive rods on a Gram stain. Similar Gram staining findings were also reported by Muranaka et al. in a catheter-related bloodstream infection caused by *M. wolinskyi* [11]. When subcultures from positive blood cultures show white, rough colonies suggestive of RGM on blood agar, prompt communication between the microbiology laboratory and attending physicians is essential to facilitate rapid species identification. It should also be recognized that mycobacteria detected in blood cultures are usually not members of the *M. tuberculosis* complex or *M. avium* complex; thus, awareness of other RGM species such as *M. wolinskyi* is critical.

In previously reported cases, treatment of *M. wolinskyi* infections typically involved both device removal and prolonged multidrug antimicrobial therapy, often for several months. As noted by El Helou et al. [12], device removal reduces relapse risk, but adequate and prolonged antimicrobial therapy remains crucial for cure. In contrast, in the present case, prolonged therapy was not feasible due to the development of CDI. Remarkably, the patient achieved complete recovery after CV port removal alone, without recurrence, and was able to undergo re-implantation of a new CV port thereafter. A plausible explanation for this favorable outcome is that the infection was confined to the port surface biofilm, with limited invasion and a small bacterial burden. Rapid removal of the device likely prevented further dissemination, and the patient’s preserved immune function may have contributed to spontaneous clearance of residual organisms. Such a biofilm-localized infection pattern has been described in other RGM, including *Mycobacterium fortuitum*, *Mycobacterium chelonae*, and *Mycobacterium abscessus*, which can adhere to synthetic materials and form resilient biofilms [12]. To our knowledge, this represents a unique clinical course that has not been previously documented.

Given the increasing use of implantable devices in elderly and immunocompromised patients, clinicians should maintain a high index of suspicion for RGM, including *M. wolinskyi*, in suspected device-related infections. Close collaboration between clinicians and microbiology laboratories remains essential for timely recognition and optimal management.

## Conclusions

*M. wolinskyi* is a rare but clinically important cause of device-associated bloodstream infection. Because this organism may appear as slender Gram-positive rods on Gram staining, awareness among clinicians and close communication with microbiology laboratories are essential for timely recognition. Confirmation by acid-fast staining and molecular or proteomic identification techniques enables accurate diagnosis. Although device removal and prolonged antimicrobial therapy are generally recommended, this case demonstrates that device removal alone can achieve a successful outcome when bacteremia resolves promptly and infection remains localized.
